# Peripheral proteomic changes after electroconvulsive seizures in a rodent model of non-response to chronic fluoxetine

**DOI:** 10.3389/fphar.2022.993449

**Published:** 2022-10-31

**Authors:** Rodolphe H. Lebeau, Indira Mendez-David, Laura Kucynski-Noyau, Céline Henry, David Attali, Marion Plaze, Romain Colle, Emmanuelle Corruble, Alain M. Gardier, Raphaël Gaillard, Jean-Philippe Guilloux, Denis J. David

**Affiliations:** ^1^ Batiment Henri Moissan, CESP-Inserm, MOODS Team, Université Paris-Saclay, Orsay, France; ^2^ PAPPSO, Micalis Institute, INRAE, AgroParisTech, Université Paris-Saclay, Jouy-en-Josas, France; ^3^ Centre Hospitalier Sainte Anne, Service Hospitalo-Universitaire, Paris, France; ^4^ Université Paris-Saclay, Faculté de Médecine, CESP-Inserm, MOODS Team, Le Kremlin Bicêtre, France; ^5^ Service Hospitalo-Universitaire de Psychiatrie de Bicêtre, Hôpitaux Universitaires Paris-Saclay, Assistance Publique-Hôpitaux de Paris, Hôpital de Bicêtre, Le Kremlin Bicêtre, France

**Keywords:** electroconvulsive therapy, fluoxetine, non-response, peripheral biomarkers, major depressive disorder, difficult to treat depression

## Abstract

Major depressive disorder (MDD) is the psychiatric disorder with the highest prevalence in the world. Pharmacological antidepressant treatment (AD), such as selective serotonin reuptake inhibitors [SSRI, i.e., fluoxetine (Flx)] is the first line of treatment for MDD. Despite its efficacy, lack of AD response occurs in numerous patients characterizing Difficult-to-treat Depression. ElectroConvulsive Therapy (ECT) is a highly effective treatment inducing rapid improvement in depressive symptoms and high remission rates of ∼50–63% in patients with pharmaco-resistant depression. Nevertheless, the need to develop reliable treatment response predictors to guide personalized AD strategies and supplement clinical observation is becoming a pressing clinical objective. Here, we propose to establish a proteomic peripheral biomarkers signature of ECT response in an anxio/depressive animal model of non-response to AD. Using an emotionality score based on the analysis complementary behavioral tests of anxiety/depression (Elevated Plus Maze, Novelty Suppressed Feeding, Splash Test), we showed that a 4-week corticosterone treatment (35 μg/ml, Cort model) in C57BL/6JRj male mice induced an anxiety/depressive-like behavior. A 28-day chronic fluoxetine treatment (Flx, 18 mg/kg/day) reduced corticosterone-induced increase in emotional behavior. A 50% decrease in emotionality score threshold before and after Flx, was used to separate Flx-responding mice (Flx-R, *n* = 18), or Flx non-responder mice (Flx-NR, *n* = 7). Then, Flx-NR mice received seven sessions of electroconvulsive seizure (ECS, equivalent to ECT in humans) and blood was collected before and after ECS treatment. Chronic ECS normalized the elevated emotionality observed in Flx-NR mice. Then, proteins were extracted from peripheral blood mononuclear cells (PBMCs) and isolated for proteomic analysis using a high-resolution MS Orbitrap. Data are available via ProteomeXchange with identifier PXD037392. The proteomic analysis revealed a signature of 33 peripheral proteins associated with response to ECS (7 down and 26 upregulated). These proteins were previously associated with mental disorders and involved in regulating pathways which participate to the depressive disorder etiology.

## 1 Introduction

Major depressive disorder (MDD) is the psychiatric disorder with the highest prevalence in the world according to World Health Organization (Liu et al., 2020). MDD can lead to significant mortality, morbidity, reductions in quality of life, and have considerable costs for the society (American Psychiatric Association 2013). The main treatments for moderate to severe MDD are based on antidepressant drugs (AD), such as selective serotonin reuptake inhibitor (SSRI). However, the pharmacotherapeutic strategy is partially efficient. Indeed, the STAR*D study (Rush et al., 2006; Trivedi et al., 2006) revealed that the remission is obtained only in one-third of treated patients with a first antidepressant family and this rate can be increased up to 70% using various pharmacotherapeutic approaches. Non-response and/or resistance occurs in a large subset of patients, constituting difficult-to treat depression, that can lead to Treatment Resistant Depression (TRD).

In order to treat these patients, many strategies have been investigated, including fast-acting therapy such as esketamine nasal spray (Bahji et al., 2021) or brain stimulation therapies (Voineskos et al., 2020). Electroconvulsive therapy (ECT), a therapeutic protocol known since 1930s for treat psychiatric disorders (Pagnin et al., 2004), is the most used and the most effective approach with a remission rate ≈80% (Group 2003) and can be applied as a second line of treatment (Milev et al., 2016).

The potential of peripheral proteome (the panel of detectable proteins) for monitoring health and disease states was investigated for brain diseases (Htike et al., 2019) including mood disorders (Preece et al., 2018). Moreover, detecting peripheral mRNA or proteins whose expression is specifically regulated by a treatment allow the identification of potential biomarkers of response to treatment that can have a predictive value (Guilloux et al., 2015; Mendez-David et al., 2017).

Several clinical studies tried to identify novel biomarkers predicting ECT outcomes and responses to targeted treatments [for review, (Maffioletti et al., 2021)]. Thus, ECT modulates the expression of 10 and ≈40 proteins in the blood of patients after either acute or chronic ECT, respectively (Stelzhammer et al., 2013). Moreover, AD treatment adjuncts with chronic ECT changes proteins profile compared to patients receiving only ECT. However, most of the studies are concentrated on one or few markers and many studies are relatively old, with small sample sizes and methodological biases (Maffioletti et al., 2021).

Many studies led during the last 30 years have shown that electroconvulsive seizure (ECS), the preclinical version of ECT, induces the expression modulation of a large variety of genes in a tissue-dependent manner (Sakaida et al., 2013; Kobayashi and Segi-Nishida 2019; Rimmerman et al., 2021). However, very few studies looked at the protein expression modulating effect of ECS. Some studies have described protein changes in the brain parenchyma (Pinna et al., 2018) or in naïve animals (Glaviano et al., 2014), which limits the use of these proteins as biomarkers of ECS and their translational utility. Thus, it will be of interest to study the effects of ECS on peripheral proteome as a surrogate for assessing treatment efficacy in a mouse model of AD non-response.

The purpose of our study will be to identify proteome variations induced by ECS in the peripheral blood mononuclear cells (PBMC), in the context of non-response to an antidepressant drug in an animal model of anxio-depressive disorders. To model the pathology, we used a pharmacological-induced model of depressive-like behavior previously developed by our team (David et al., 2009; Mendez-David et al., 2017). Concretely, mice were treated first with corticosterone (Cort) to induce a depressive-like phenotype, then with a combination of Cort and fluoxetine (Flx) for 4 weeks. After each step of treatment, an emotionality score (equivalent to clinical scores assessing the severity of depression) was established (Guilloux et al., 2011) to evaluate changes in emotional behavior and treatment response. After Flx treatment, mice that failed to recover a score similar to non-depressed mice receive a 2-weeks ECS treatment. A blood draw was performed in animals before and after ECS treatment for proteomic analysis, revealing a signature of 33 peripheral proteins associated with response to ECS (7 down- and 26 upregulated), with one priorly found to be associated to antidepressant response (Mendez-David et al., 2017).

## 2 Materials and methods

### 2.1 Animals

Adult C57BL/6JRj male mice were purchased from Janvier Farms (Le Genest St Isle, France). All mice were 7–8 weeks old, weighed 23–25 g at the beginning of the treatment and were maintained on a 12L:12 D schedule (lights on at 06:00). Mice were housed in groups of five. Food and water were provided *ad libitum*. The protocols involving animals and their care were conducted in conformity with the institutional guidelines in compliance with national and international laws and policies (Council directive #87–848, 19 October 1987, Ministère de l’Agriculture et de la Forêt, Service Vétérinaire de la Santé et de la Protection Animale, permissions # 92–256B to DJD) and in compliance with protocols approved by the Institutional Animal Care and Use Committee (CEE26 authorization #4747).

### 2.2 Treatments

Corticosterone (4-pregnen-11b-DIOL-3 20-DIONE 21-hemisuccinate from Sigma (Sigma-Aldrich Saint-Quentin Fallavier, France) was dissolved in vehicle (0.45% hydroxypropyl-β-cyclodextrin, Sigma-Aldrich Saint-Quentin Fallavier, France). Fluoxetine hydrochloride (18 mg/kg per day in the drinking water) was purchased from Anawa Trading (Zurich, Switzerland).

### 2.3 Protocol

The dose and duration of corticosterone treatment were selected based on previous study [Cort model (David et al., 2009; Mendez-David et al., 2013; Mendez-David et al., 2014),]. Corticosterone (35 μg/ml, equivalent to about 5 mg/kg/day, *n* = 31) or vehicle (0.45% β-cyclodextrine, β-CD, *n* = 13) were available *ad libitum* in the drinking water during the first 4 weeks of the protocol (Supplementary Figure S2A).

**FIGURE 1:**
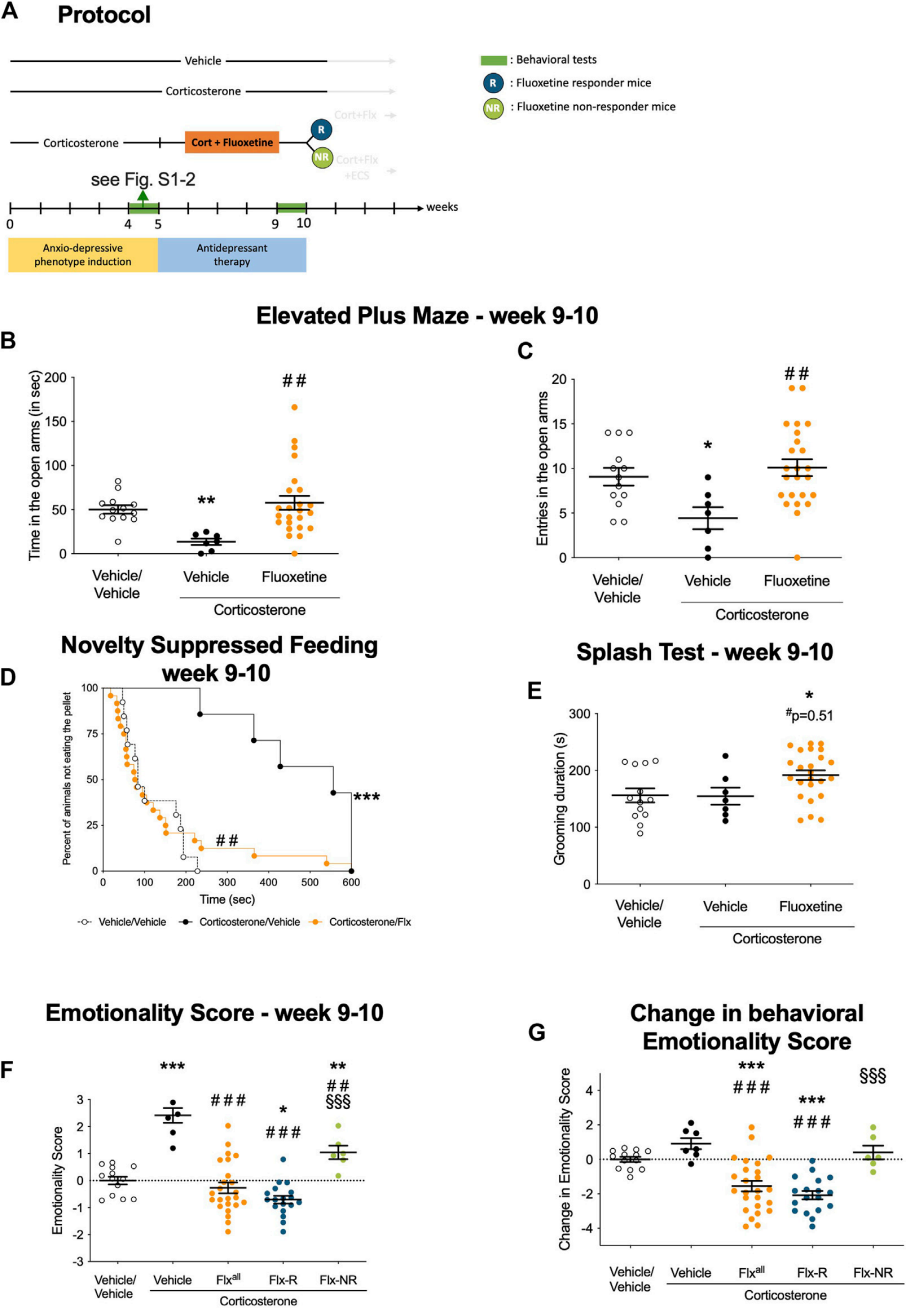
Chronic fluoxetine treatment produces reversed anxiety and depression-like phenotype in a mouse model of anxiety/depression. **(A)** Timeline of experiments: In place of normal drinking water, grouped-housed male C57BL/6JRj mice were presented during 10 weeks with vehicle (0.45% hydroxypropyl-β-cyclodextrin) or corticosterone (35 μg/ml) in the presence or absence of an antidepressant (fluoxetine, 18 mg/kg/day) during the last 5 weeks of the corticosterone regimen. Emotionality z-score was calculated after each behavioral session. Then, we investigated whether the behavioral changes induced after chronic corticosterone (week 4–5, see Supplementary Figure S2) were reversed by fluoxetine treatment (week 9–10, Figures 1B–G). The same animal was successively tested in the Elevated Plus Maze (EPM), the Novelty Suppressed Feeding (NSF), the Splash Test (ST) during both behavioral sessions **(B–C)** Effects of corticosterone (35 μg/ml, Cort) regimen given alone or in combination with fluoxetine (18 mg/kg/day) on anxiety behaviors measured at Week 9–10 in the Elevated Plus Maze (EPM). Anxiety, measured for various parameters is expressed as mean total time in seconds **(B)** or entries **(C)** in open arms of EPM paradigm.**(D)** Effects of 4 weeks of corticosterone regimen (35 μg/ml) alone or in combination with fluoxetine (18 mg/kg/day) on anxiety- and depression related behaviors measured at Week 9–10 in the Novelty Suppressed Feeding paradigm. Results are expressed as cumulative survival with percentage of animals that have not eaten over 10-min. **(E)** Effects of 4 weeks of corticosterone regimen (35 μg/ml) alone or in combination with fluoxetine (18 mg/kg/day) on depression related behaviors in the Splash Test (ST) measured at Week 9–10. Results are expressed as mean of grooming duration (in seconds). **(F-G)** Effects of 4 weeks of corticosterone regimen (35 μg/ml, Cort) alone or in combination with fluoxetine (18 mg/kg/day) on anxiety/depression-like behaviors on the emotionality score measured at Week 9–10. Test Z-values (elevated plus maze, novelty-suppressed feeding and splash test) are calculated by averaging individual Z-scores to obtain emotionality Z-scores **(F)**. Change in behavioral emotionality score between W4 and W10 **(G)**.Values plotted are mean ± SEM [*n* = 7–21 animals for vehicle (VEH, open circle), corticosterone (Cort, black dot), corticosterone-fluoxetine mice (Fluoxetine or Flx^all^, orange dot) corticosterone-fluoxetine responding mice (Flx-R, blue circles), corticosterone-fluoxetine non-responding mice with ECS treatment (Flx-NR ECS, green circles)]. One way ANOVA with post-hoc tests or Kaplan–Meier survival analysis followed by Mantel–Cox log-rank test were applied (*p < 0.05, **p < 0.01 versus Vehicle/vehicle group, #*p* < 0.05, #*p* < 0.01 versus Cort/vehicle group, §*p* < 0.05, §§*p* < 0.01 versus Flx-R mice).

Chronic corticosterone consumption in mice dramatically inhibit the HPA axis function and response to stress with a resulting low concentration of serum corticosterone (See Supplementary Figure S3E, David et al., 2009) During the following 4 weeks of the protocol, corticosterone was delivered alone (*n* = 7 animals) or in the presence of fluoxetine (18 mg/kg/day, *n* = 24 animals, Figure 1A). Treatments were maintained until the end of the experiments. Behavioral sessions to assess anxiety/depression-like phenotype, the antidepressant response to fluoxetine and to chronic ECS occurred on week 5, 10, and 12, respectively. Thus, each animal underwent three behavioral sessions aiming at evaluating emotional behavior.

### 2.4 Electroconvulsive seizure

**FIGURE 2:**
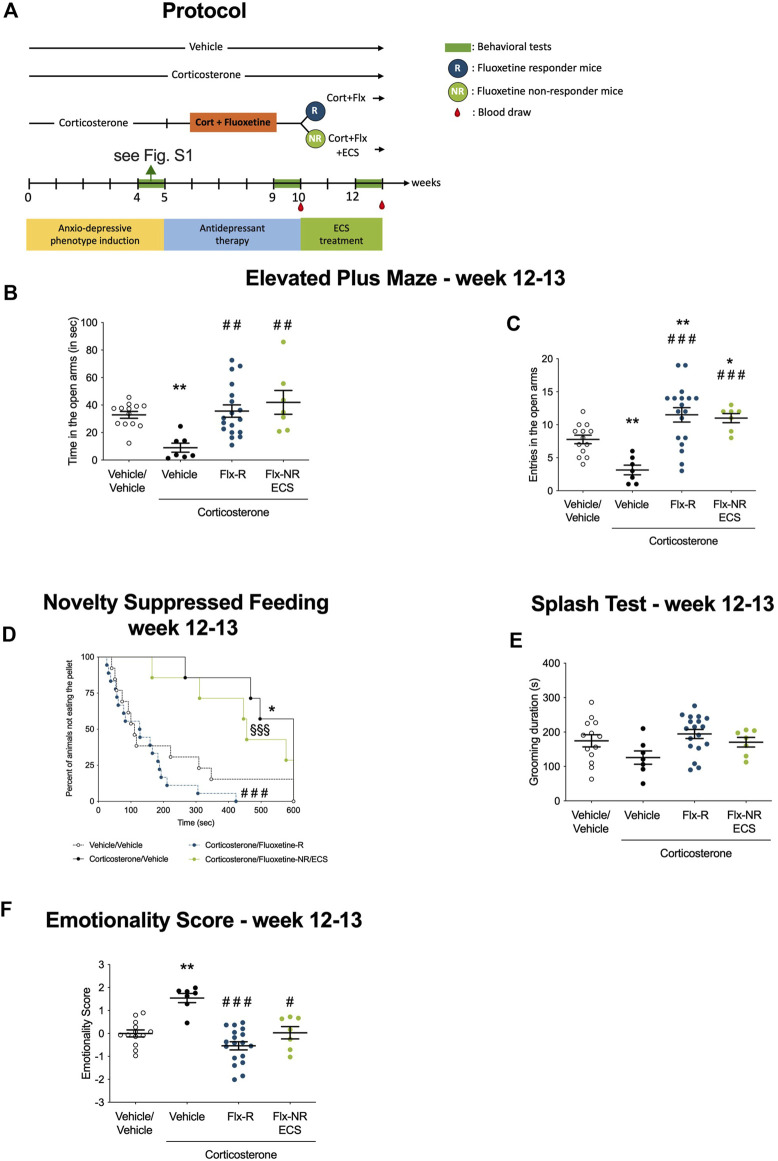
Fluoxetine non-responding mice emotional behavior was rescued after chronic electroconvulsive seizures (ECS administration). **(A)** Timeline of experiments: Following protocol presented in Figure 1A, Responders and non-responders animals were selected and Peripheral Blood Mononuclear Cells were isolated from whole blood after W10. Chronic ECS treatment was applied in NR-mice while R underwent sham treatment. Emotional behavior was tested at W12 after ECS administration and PBMC extracted from whole blood. **(B–C)** Effects of chronic ECS on anxiety behaviors in the Elevated Plus Maze (EPM) in Flx-NR mice. Anxiety, measured for various parameters is expressed as mean total time in seconds **(B)** or entries **(C)** in open arms of EPM paradigm. **(D)** Effects of chronic ECS on anxiety- and depression related behaviors in the Novelty Suppressed Feeding paradigm in Flx-NR mice. Results are expressed as cumulative survival with percentage of animals that have not eaten over 10-min. **(E)** Effects of 4 weeks of chronic ECS on depression related behaviors in the Splash Test (ST). Results are expressed as mean of grooming duration (in seconds). **(F)** Effects of 4 weeks of chronic ECS on the emotionality score. Test Z-values (elevated plus maze, novelty-suppressed feeding and splash test) are calculated by averaging individual Z-scores to obtain emotionality Z-scores **(F)**.Values plotted are mean ± SEM [*n* = 7–21 animals for vehicle (VEH, open circle), corticosterone (Cort, black dot), corticosterone-fluoxetine responding mice (FLX-R, blue dots), corticosterone-fluoxetine non-responding mice with ECS treatment (FLX-NR ECS, green dots)]. One way ANOVA with post-hoc tests or Kaplan–Meier survival analysis followed by Mantel–Cox log-rank test were applied (*p < 0.05, **p < 0.01 versus Vehicle/vehicle group, #*p* < 0.05, ##*p* < 0.01 versus Cort/vehicle group, §*p* < 0.05, §§*p* < 0.01 versus Flx-R mice).

The ECS paradigm consisted of seven ECS sessions across a 15-days period (once every 2 days, Figure 2A) delivered with an Ugo Basile pulse generator (model #57800–001, shock parameters: 100 pulse/s frequency, 3 ms pulse width, 1 s shock duration and 50 mA current). Mice were administered inhaled isoflurane anesthesia (2%) prior to ECS sessions, and they remained anesthetized throughout the procedure. The stimulation parameters were chosen because they reliably induced tonic-clonic convulsions (Schloesser et al., 2015). Fluoxetine treatment was still administered during the 2 weeks of ECS treatment.

### 2.5 Behavioral experiment evaluation

#### 2.5.1 Elevate maze plus

The elevated plus maze (EPM) is a widely used behavioral assay for rodents and it has been validated to assess the anti-anxiety effects of pharmacological agents [for review (Walf and Frye 2007)]. This test was performed as described previously (Mendez-David et al., 2014). The maze is a plus-cross-shaped apparatus, with two open arms and two arms closed by walls linked by a central platform 50 cm above the floor. Mice were individually put in the center of the maze facing an open arm and were allowed to explore the maze for a duration of 5 min. The time spent in and the numbers of entries into the open arms were used as an anxiety index. All parameters were measured using a videotracker (EPM3C, Bioseb, Vitrolles, France).

#### 2.5.2 Novelty suppressed feeding test

The NSF is a conflict test that elicits competing motivations: the drive to eat and the fear of venturing into the center of a brightly lit arena. The latency to begin eating is used as an index of anxiety/depression-like behavior, because classical anxiolytic drugs as well as chronic antidepressants decrease this measure. The NSF test was carried out during a 10 min period as previously described (David et al., 2009). Briefly, the testing apparatus consisted of a plastic box (50 × 40 × 20 cm), the floor of which was covered with approximately 2 cm of wooden bedding. 24 h prior to behavioral testing, all food was removed from the home cage. At the time of testing, a single pellet of food (regular chow) was placed on a white paper platform positioned in the center of the box. Each animal was placed in a corner of the box, and a stopwatch was immediately started. The latency to eat (defined as the mouse sitting on its haunches and biting the pellet with the use of forepaws) was timed. Immediately afterwards, the animal was transferred to its home cage, and the amount of food consumed by the mouse in the subsequent 5 min was measured serving as a control for change in appetite as a possible confounding factor.

#### 2.5.3 Splash test

This test consisted of squirting a 10% sucrose solution on the mouse’s snout. This procedure induces grooming behaviors, due to the viscosity and palatability of the sucrose. The grooming behavior is sensitive to chronic stress or chronic Cort exposure and antidepressant treatment (Mendez-David et al., 2014). The total time spent in different grooming behaviors (i.e., face, paws, hindquarter, and shoulders) was directly recorded for 5 min in the home cage of the animals.

#### 2.5.4 Behavioral emotionality measurement

Three behavioral tests (i.e., EPM, NSF and ST) were used to measure components of animal behavioral emotionality. Z-score methodology was used to investigate the potential of combining results within and across the different behavior tests for depressive/anxious-like behaviors and investigate the treatment effects in the Cort model. The emotionality-related data was normalized as previously described (Guilloux et al., 2011; Mekiri et al., 2017). Briefly, z scores are standardized scores (by the group mean and group standard deviation). They indicate how many standard deviations (σ) an observation (x) is above or below the mean of a control group (µ).

$$z=\frac{X-\mu }{\sigma }$$

Z scores for behavioral measures were first averaged within the test, and then across the test for equal weighting of the three tests comprising the final emotionality score. The increased behavioral emotionality was defined as decreased activity in the open arms in the EPM, increased NSF latency and decreased grooming in the splash test compared with control group means. The vehicle group was defined as the control. Emotionality score was calculated after each behavioral round.

### 2.6 Isolation of mouse peripheral blood mononuclear cells

To determine a biological signature of response to ECS, representative animals of each group were used for proteomics analysis. The procedure was performed in unanesthetized mice as previously described (Mendez-David et al., 2013). In compliance with the laboratory animal care guidelines, about 0.4 ml of blood per mice was collected in K_3_EDTA tubes using the submandibular bleeding method. The punctures were performed with 5 mm point size sterile lancets (MediPoint, Mineola, NY) where the orbital vein and the submandibular vein join to form the jugular vein (Joslin 2009). A light pressure with dry gauze was applied to the punctured area for hemostasis. Separation and extractions of PBMCs were done using the iodixanol mixer technique (Ford and Rickwood 1990). Separations of mouse PBMCs were purified of mouse whole blood through density centrifugation (1,000 rpm at 20°C for 30 min) using solution B with the OptiPrep™ gradient solution (Sigma-Aldrich Saint-Quentin Fallavier, France). After centrifugation, OptiPrep™ gradient solution separated layers of blood, with PBMCs under a layer of plasma. The PBMCs layers were carefully removed from the tube and transferred to a new 50 ml conical tube and were washed twice with solution B. After centrifugations (1,200 rpm at 20°C for 7 min) and several washing steps, mouse PBMCs were recovered with a last centrifugation (3,000 rpm at 4°C for 5 min) and stored at 80°C before subsequent assay.

### 2.7 Proteomics analysis

#### 2.7.1 Protein separation

Protein extracts from PBMCs were homogenized in solution solubilization (Urea 7M, Thiourea 2M, CHAPS 3%, Nonidet P-40 1%, DTT 1%). Protein concentration was measured using 2D-Quant kit (GE Healthcare, France) and 15 µg of proteins were loaded and separated by 12% SDS-PAGE. A short migration was then performed (7 min, 80 V, 25 W followed by 4 min, 200 V, 25 W) and gels were stained with Coomassie colloidal blue (EZblue, Sigma-Aldrich, France).

#### 2.7.2 Protein in-gel digestion

Portions of gel that contain all proteins were cut and digested as followed: pieces of gel were successively washed and de-stained with water, acetonitrile (ACN) and 25 mM ammonium bicarbonate (NH_4_HCO_3_). A reduction/alkylation step was performed with dithiothreitol (DTT) 10 mM and iodoacetamide 55 mM. Gels were dehydrated with acetonitrile and rehydrated at 4 °C in 12 ng/μl sequencing grade modified trypsin (Promega, France) solubilized in 25 mM NH_4_HCO_3_ in 1 h and then digested at 37°C overnight. After tryptic digestion, peptides were extracted by incubating gel pieces in extraction solvent (0.5% trifluoroacetic acid (TFA)/50% ACN) for 15 min and in ACN for 15 min at room temperature. Supernatants were vacuum dried. The dried extract peptides were dissolved in 50 μl of loading buffer (0.08% TFA/2% ACN) just before mass spectrometry analysis.

#### 2.7.3 Mass spectrometry analysis

Four microliters of sample were loaded on the nano-UPLC Ultimate 3000 RSLCnano (Thermo). Sample was loaded at 20 μL/min on the pre-column cartridge (PepMap 100 C18, 5 µm; 300 µm i.d., 5 mm, Thermo Scientific) and peptides were then separated with a gradient of acetonitrile on the reverse phase column PepMap 100 C18 (stationary phase: C18, 3 μm; column: 75 µm i.d., 500 mm; nanoViper, Thermo Scientific, France). Buffers were 0.1% formic acid in 2% acetonitrile (A) and 0.1% formic acid in 80% acetonitrile (B). The peptide separation was realized during 64 min at 300 nL/min with a linear gradient from 0 to 45% B for 55 min followed by a gradient from 45% to 98% B for 5 min. Eluted peptides were analyzed on-line with a high resolution mass spectrometer Orbitrap Fusion Lumos Tribrid (Thermo Scientific, France) using a nanoelectrospray interface in positive polarity mode, on PAPPSO platform (http://pappso.inra.fr). Peptide ions were analyzed using Xcalibur 3.0 (Thermo Scientific, France) with following data-dependent acquisition steps: 1) full MS scan in orbitrap (mass-to-charge ratio [*m*/*z*] = 400–1500; mass tolerance, ± 10 ppm) and 2) MS/MS in Ion Trap with CID activation (collision energy, 35%; activation time, 30 ms; centroid mode). Dynamic exclusion time was set to 60 s.

### 2.8 Statistical analysis

#### 2.8.1 Behavioral analysis

To assess the behavioral consequences of a chronic corticosterone treatment in the EPM, NSF, and ST tests or emotionality scores, results were expressed as mean ± SEM values. Normality of data were checked using a Shapiro-Wilk normality test. Depending on the normality of the data, the parametric or non-parametric procedure was performed using a Student *t*-test or a Mann and Whitney test for two groups’ comparison. For analysis with >2 groups, a one-way ANOVAs or a Kruskal–Wallis test were applied to the data as appropriate. Significant main effects were followed by Fisher’s *post-hoc* or Dunn’s multiple comparison tests. Regarding the NSF test, we used the Kaplan–Meier survival analysis owing to the lack of normal distribution of the data. Mantel–Cox log rank test was used to evaluate differences between experimental groups. Statistical significance was set at *p* < 0.05. Data were analyzed using Prism 8.4.3 software (GraphPad, La Jolla, United States).

#### 2.8.2 Data processing and bioinformatics analysis

Peak lists were generated as mzXML files using the converter MSConvert (ProteoWizard). A database search was performed using X!TandemPipeline software developed by PAPPSO facility (version 3.4.3; http://pappso.inra.fr/bioinfo/xtandempipeline/.3; http://pappso.inra.fr/bioinfo/xtandempipeline/) (Langella et al., 2017) with search parameters as followed: enzymatic cleavage by trypsin digestion with one possible miscleavage, fixed carbamido-methylation modification on cysteine and variable oxidation on methionine; precursor mass tolerance of ± 10 ppm and fragment mass tolerance of 0.5 Da. Several databases were used: the Uniprot KB/SwissProt Mus musculus database (24,977 entries, version January 2017) and a homemade contaminant database (trypsin, keratine, *etc.*). The identified proteins were filtered with a minimum of two different peptides required with a peptide *E-value* < 0.01, and a protein *E-value* (product of unique peptide *E values*) < 10^–4^. Combine analysis mode with all samples was performed and results were grouping proteins: proteins which have at least one peptide in common. This allowed to group proteins with similar functions. Within each group, proteins with at least one specific peptide relatively to other members of the group were reported as subgroups. One subgroup represents one specific protein. Proteins are characterized with their spectral number. Label free quantification of proteins were achieved with spectral counting approach (SC), which is a strategy to determine a relative quantification of proteins from their number of spectra obtained with tryptic peptides in MS. This quantification relies on the more of a particular protein is present in a sample, the more MS spectra are detected for peptides of that protein. Statistical analysis was performed using MassChroqR package developed by PAPPSO team (http://pappso.inra.fr/bioinfo/masschroq/) (R version 3.3.2). A generalized linear mixed model (GLM) with a Poisson distribution was applied. This model suits in the case of a counting like SC. The principal component analysis was obtained by simulating the kernel densities from group’s means and variances assuming bivariate normal distributions. This distribution was generated using protein abundances as variables. Hierarchical bivariate clustering was performed using Euclidean distances and unweighted pair group averages as the aggregation method. All data analyses and graphical representations were performed using the R package MassChroq. Significant changes in protein abundance was determined by analysis of variance (ANOVA) using a Chi-square test. Treatment effect was considered with an adjusted *p* value for multiple testing by a Benjamini–Hochberg procedure (Benjamini and Hochberg 1995). Student *t* tests were performed to identify proteins which showed significant differences expressed between groups with the following criteria: *p*-value was set at <0.05. The resulting exploratory list may carry a higher rate of false positives at the protein level but allowed investigation of cumulative effects over larger sets of proteins and pathways.

#### 2.8.3 Ingenuity pathway analysis

Selected proteins were overlaid on the global molecular network of Ingenuity Pathway Analysis (Ingenuity^®^ Systems, www.ingenuity.com) allowing for a generation of gene networks based on their connectivity. Their score takes into account the relative numbers of network eligible molecules, of molecules analyzed and the total number of molecules in Ingenuity’s knowledge base. Disease links are generated on the literature-based association with illness by IPA.

## 3 Results

Detailed statistical results are provided in Supplementary Table S1.

### 3.1 Physiological response to treatments

As previously observed (David et al., 2009), a 4-week treatment with Cort increased mouse body weight in comparison to controls (Supplementary Figure S1A, *p* < 0.01). Chronic Flx treatment decreased weight gain compared to Cort or vehicle-treated mice (Supplementary Figure S1B, *p* < 0.001 and *p* < 0.01, respectively). At the end of the protocol (W12), the weight gain in the Vehicle/Vehicle mice was bigger than that in Cort/Vehicle and Cort + Flx-R mice (Supplementary Figure S1C, *p* < 0.05 and *p* < 0.01 respectively). Flx-NR mice treated with ECS showed a greater weight gain compared to Cort/Veh and Cort/Flx-R mice (*p* < 0.05 and *p* < 0.01 respectively).

### 3.2 Chronic corticosterone induced higher emotionality

A 4-week treatment with Cort (Supplementary Figure S2A) induced an anxiety/depression-like phenotype in C57BL/6JRj mice as previously shown: we confirm here that the higher emotionality induced by chronic corticosterone administration is stable over time throughout the entire protocol and allow for longitudinal studies (David et al., 2009; Mendez-David et al., 2014; Siopi et al., 2016; Mendez-David et al., 2017; Cabeza et al., 2021). In the EPM, Cort-treated mice displayed a decrease in the time spent and number of entries in the open arms (Supplementary Figures S2B–C, *p* < 0.01). In the NSF tests, a decrease in latency to feed was observed after chronic Cort treatment (Supplementary Figure S2D, *p* < 0.0001) that was not correlated with food consumption in the homecage (Supplementary Table S1), as previously described (David et al., 2009; Mendez-David et al., 2017; Pinna et al., 2018). No significant effect was observed in the Splash test (Supplementary Figure S2E), but the behavioral emotionality score was significantly increased after chronic Cort treatment (Supplementary Figure S2F, *p* < 0.0001) compared to the Vehicle/Vehicle treatment.

### 3.3 Selection of responders/non-responders to chronic fluoxetine treatment

A 4-week treatment with fluoxetine (18 mg/kg/d, protocol in Figure 1A) or vehicle was administered to Cort-treated mice and antidepressant response was monitored with the same battery of tests as in Supplementary Figure S2.

In the EPM, Cort/Flx mice showed an increase in the time spent (Figure 1B, *p* < 0.01) and the number of entries in the open arms of the EPM (Figure 1C, *p* < 0.01) compared to Cort/Veh mice. In the NSF, a 4-week Flx treatment reduced the increase in the latency to feed after Cort (Figure 1D, *p* < 0.01). A non-significant (*p* = 0.51) increase in grooming duration was observed in Cort/Flx *versus* Cort/Veh mice in the splash test (Figure 1E). Applying z-normalization across tests after the second round of behavior showed that chronic Flx decreased Cort-induced emotionality score (Figure 1F, *p* < 0.0001 *versus* Veh/Veh group; *p* < 0.0001 *versus* Cort/Veh group). Thus, chronic Flx decreased anxiety/depression-like phenotype in Cort-treated mice, confirming prior observations (Guilloux et al., 2011; Mendez-David et al., 2015). Overall, chronic Flx treatment significantly reduced behavioral emotionality in Cort-treated mice, as shown in measuring the change in emotionality score value between Week 10 and Week 5 (Figure 1G, *p* < 0.001).

As previously shown (Mendez-David et al., 2017), phenotypic variability was observed after chronic Flx administration. Two subgroups of mice could be defined based on the reduction of their emotionality score: a 50% decrease in emotionality score threshold was used to separate Flx-responding mice (Flx-R, *n* = 18), or Flx non-responders mice (Flx-NR, *n* = 7). The 50% threshold was defined for each animal individually, by comparing their individual score at W10 vs. the score obtained at W5. This threshold was based on the clinical human criterion (Nierenberg and DeCecco 2001) and previously used in preclinical studies (Mendez-David et al., 2017). These two groups displayed a significant difference in behavioral emotionality levels (Figure 1G, *p* < 0.0001).

### 3.4 Effect of chronic electroconvulsive seizures treatment in fluoxetine non-responding mice

We tested the effects of chronic ECS in Flx-NR mice who received seven sessions of ECS over 14 days, while Flx treatment was maintained in the two groups of Flx-treated mice (R and NR) as described in the protocol (Figure 2A).

In the EPM, ECS in Flx-NR mice increased time spent and number of entries in open arms compared to Cort/Veh mice (Figures 2B,C, *p* < 0.01 and *p* < 0.001, respectively). By contrast, EPM parameters in Flx-NR mice treated with ECS were not significantly different from those measured in Flx-R mice (Figures 2B,C, *p* = 0.387 and *p* = 0.742, respectively). In the NSF test, chronic Flx treatment in responder animals was still effective in comparison to Cort/Veh mice (Figure 2D, *p* < 0.0001). Non significant decrease in the latency to feed in the NSF and increase the grooming behavior in the splash test were observed in the Cort/Flx-ECS group in comparison to the Cort/Veh group (Figure 2D, *p* = 0.26; Figure 2E, *p* = 0.14). Overall, we observed that ECS reduced the emotionality score in Flx-NR mice as indicated by its significant decreased value compared to Cort/Veh (Figure 2F, *p* < 0.05). Moreover, no relevant difference between the Flx-R and Flx-NR-ECS groups was observed (Figure 2F, *p* = 0.14).

### 3.5 Protein changes in Flx-NR mice after chronic electroconvulsive seizures treatment

**FIGURE 3:**
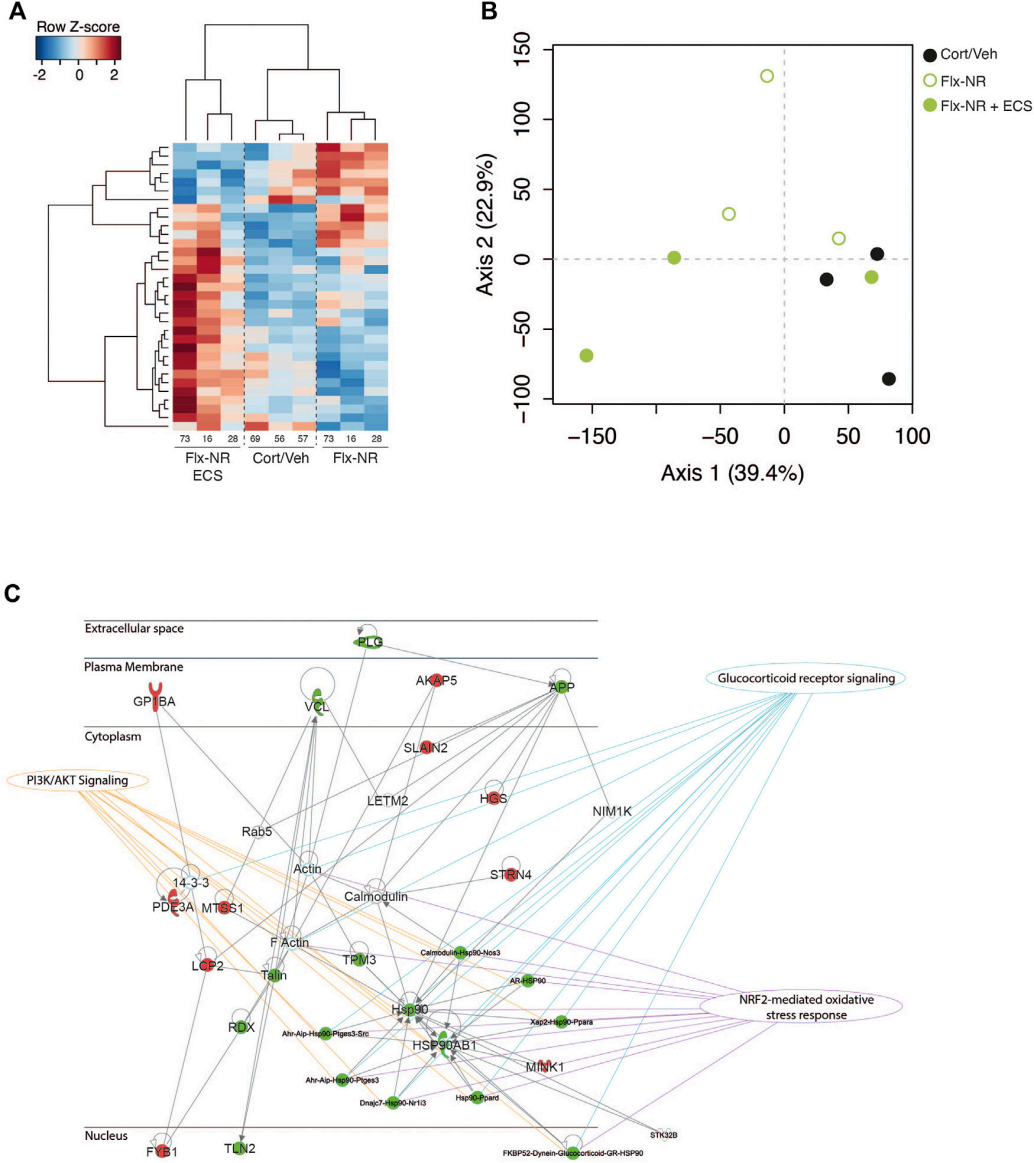
Peripheral proteomic changes after fluoxetine exposure in responders and non-responders **(A)** Hierarchical bivariate clustering of expression profiles of animals (column) and proteins (rows) depicts the differences between Cort/Veh, Flx-NR, and Flx-NR-ECS groups. An animal’s expression is red for above-average values, and blue for below-average values **(B)** Principal Component Analysis of expression profiles revealed two main axis separating results. **(C)** Ingenuity Pathway Analysis revealed a molecular interaction network based on differentially expressed proteins. This protein network was significantly connected to the following canonical pathways: glucocorticoid receptor signaling, PI3K/Akt signaling and NRF2-mediated oxidative stress response.

Using a high resolution mass spectrometry analysis by X!TandemPipeline, specific proteins in PBMCs (*n* = 3 samples per group) were detected (Supplementary Tables S2). Characterized proteins with less than two peptides were excluded. Hierarchical clustering of the expressed proteins distinguished Cort/Veh treated mice from Cort/Flx-NR and Cort/Flx-ECS mice (Figure 3A) and revealed 33 proteins showing differential changes. This aggregate behavior of this large-scale systemic response was quantified with Principal Components Analysis (PCA, Figure 3B), which confirmed hierarchical clustering analysis. According to PCA, proteins’ abundance separated Cort/Veh, Cort/Flx-NR and Cort/Flx-ECS mice.

A group effect was observed for 33 proteins (*p* < 0.05, Supplementary Table S2). The levels of expression of 14 proteins were significantly modified by chronic fluoxetine treatment in Cort/Flx-NR mice, and 17 proteins in Cort/Flx-ECS mice compared to Cort/Veh mice (*p* < 0.05, Figure 3A and Supplementary Table S2). ECS induced significant changes in expression of 18 proteins (7 down-regulated and 11 upregulated).

Ingenuity Pathway analysis revealed three main networks associated with the 33 differentially expressed proteins (Figure 3C; Table 1), which relates to broad diseases and disorders such as “Inflammatory response,” “Cardiovascular or Hematological Diseases.” Canonical pathways linked to these DE proteins evidently relates to circulating components or process found in blood.

**TABLE 1:** Ingenuity pathway analysis results.

Top Networks		Score
1) Cell Morphology, Hematological System Development and Function, Inflammatory Response		43
2) Cellular Development, Cellular Growth and Proliferation, Hematological System Development and Function		31
3) Cancer, Cell Morphology, Cellular Movement		3
Top diseases and Disorders	# Molecules	*p-value*
Inflammatory Response	13	4.01E-03 - 4.55E-08
Cardiovascular Disease	8	4.01E-03 - 6.15E-07
Hematological Disease	18	4.01E-03 - 6.15E-07
Organismal Injury and abnormalities	29	4.01E-03 - 6.15E-07
Connective Tissue Disorders	13	4.01E-03 - 1.26E-05
Top canonical pathways	−log (*p-value*)	Ratio
Fc Receptor-mediated Phagocytosis in Macrophages and Monocytes	3.6	0.032 (3/94)
Neuroprotective Role of THOP1 in Alzheimer's Disease	3.3	0.025 (3/118)
Leukocyte Extravasation Signaling	2.7	0.016 (3/193)
Remodeling of Epithelial Adherens Junctions	2.4	0.029 (2/68)
Actin Cytoskeleton Signaling	2.4	0.012 (3/245)

Results show top biological networks, diseases and disorders and canonical pathways obtained by Ingenuity Pathway Analysis (IPA). Network scores represent negative log-values of right-tailed Fisher’s Exact Tests for network consistence. Pathways and disease *p*-values represent significance of over-representation of candidate proteins within respective protein groups. *p*-value ranges indicate values for various disease sub-classifications.

## 4 Discussion

### 4.1 Electroconvulsive seizures corrected anxiety/depression phenotype in Flx-NR

Lack of treatment response is one of the major limits of current antidepressant treatment, which can be circumvent using pharmacological combination or treatment. Novel pharmacological approaches including ketamine are also gaining attention, however they yet did not present a superiority of effect compared to electroconvulsive therapy (Veraart et al., 2021). Despite the frequent and widespread use of ECT, possible cognitive impairments and other adverse effects were described and its molecular mechanisms underlying its efficacy remains largely unclear [for review (Maffioletti et al., 2021)]. Thus, before engaging into ECT treatment, the search for predictors of ECT response in animal models of anxiety/depression would benefit to patients with MDD.

This work was performed in a different and new cohort of mice compared to our previous report on peripheral markers associated with non-response to Flx (Mendez-David et al., 2017). Here, we confirmed our previous report showing non-response to chronic fluoxetine (Flx-NR) treatment in a pharmacological model of anxiety/depression (Mendez-David et al., 2017). We found a ≈72% response rate to chronic fluoxetine (18 responders out of 25 Flx-treated mice). This response rate is very similar to our prior work (65%, Mendez-David et al., 2017), performed with the same experimental protocol, with similar behavioral evaluation and threshold for defining treatment response. These preclinical results are also in line with clinical observation from the STAR*D study (Rush et al., 2006; Trivedi et al., 2006) showing ineffective antidepressant drug treatment despite a mean of several weeks of SSRI therapy.

Interestingly, we reveal that chronic ECS treatment in Flx-NR mice rescued the behavioral emotionality score. While numerous studies explored the effect of chronic effects of ECS in naïve rodents (Duman and Vaidya 1998), few of them explored its benefits and its mechanism of action in animal model of anxiety/depression (Schloesser et al., 2015; Jonckheere et al., 2018). To our knowledge, the present study is the first examining ECS effects in the context of non-response to an antidepressant drug treatment. Prior works in the Cort model of depressive disorder showed that chronic ECS reverses Cort-induced depressive-like phenotype (Schloesser et al., 2015), an effect that may be supported by both rescuing Cort-induced deficit in dendritic spine morphology and increasing expression of *Bdnf* activity-dependent exons 1 and 6 (Maynard et al., 2018). Recently, increased synaptic connectivity and extended neuronal survival were confirmed as key factors for ECS efficacy in a genetic animal model relevant to some aspects of depression (Jonckheere et al., 2018). Here, a 14-day protocol of ECS alleviated the non-response to chronic fluoxetine treatment in a neuroendocrine-based rodent model of MDD and antidepressant treatment resistance. Our preclinical ECS results confirm that the use of ECT in the context of non-response to classic antidepressant treatment could benefit to patients with MDD.

### 4.2 Peripheral proteomic changes after electroconvulsive seizures

According to a recent review on molecular biomarkers of ECT effects, most of the studies are concentrated on one or few markers. Expression studies on gene transcripts and microRNAs are rare and genetic studies are sparse. To date, no conclusive evidence regarding ECT molecular markers of clinical response has been reached (Maffioletti et al., 2021).

Our study is also the first report of ECS-induced proteome variations in the blood, and most especially in peripheral blood mononuclear cells (PBMC), samples easy to transpose into future clinical trials. The identification of protein biomarkers indicates that these proteins are involved in cell adhesion/motility (FYB1, TLN2), cell structure and cytoskeleton (CRAD, MTSS1, and SMTN), coagulation (F11R, PLG) and immune system (LCP2 and PPP6R3), signaling pathway (AKAP5, MINK1, and TMP3), vesicle trafficking (i.e., SH3GLB2, CC2D1B, and CAVIN1) or in cell metabolism (ADSS, GK, HSP90AB1, and HSG) and cell cycle (FYB1 and PPP6R3). At a single molecular level, some of these proteins have been prior associated with mental health. For example, ADSS polymorphism or increased blood levels of ADSS were associated with schizophrenia and bipolar disorder (Zhang et al., 2008a; Zhang et al., 2008b) and (Tsuang et al., 2005), respectively.

The scaffolding protein AKAP5 (also called AKAP150 in rodent species and AKAP79 on human) have a dual role in depression. Indeed, genetic blockade of AKAP5 expression in the basolateral nuclei of the amygdala prevents chronic stress -induced depressive like disorders in mice (Zhou et al., 2019). Conversely, the disruption of AKAP5 in E18 embryonic cortical neurons (Mari et al., 2015) (Mari et al., 2015) negatively affect the inhibition of ASIC1a -a ion channel known to be involved in neuropathologies including depression and whose pharmacologic inhibition has antidepressant-like effects (Coryell et al., 2009). These opposite roles in the biological processes of depression could be explain by the protein partner bound by AKAP5: the complex of AKAP5 with calcineurin inhibit the ASIC1a functions (Mari et al., 2015) while the association with PKA promote ASIC1a activity (Chai et al., 2007).

Overexpression of APP in mice has been associated with anxious and depressive-like phenotype in APP knock-in mice (Locci et al., 2021). However, conflicting reports in mouse models (Sakakibara et al., 2018; Latif-Hernandez et al., 2019) suggest a dynamic emotional response depending on the test performed (Pervolaraki et al., 2019), the age of the animal but independent of the Aβ plaque load. This last, formed by the cleavage of APP, was identified recently in cerebrospinal fluid, with other protein biomarkers, as an hypothetic biomarker of the ECT efficacy (Kranaster et al., 2019).

CC2D1B participate in a dual repressor element with Freud2, both molecules acting as strong repressors of the expression of serotonin 1A receptor, a key receptor involved in pathophysiology of depression and antidepressant drug response (Samuels et al., 2016).

While no direct link between plasminogen (PLMN) and depression has been observed, its activation into plasmin is controlled by tissue-type plasminogen activator (tPA) which cleave PLMN into plasmin. One of the role of plasmin is to convert pro-BDNF into mature BDNF in the brain, and thus this pathway has been implicated in depression and treatment response (Tsai 2017). Here, we found a reduction in PLMN levels after ECS treatment: this result correlates with prior observations showing an increase in tPa expression after ECS exposure (Segawa et al., 2013) suggesting an increase in plasminogen cleavage and increase in plasmin levels.

Prolyl endopeptidase (PPCE or PREP) peripheral levels have been observed decreased in MDD subjects (Maes et al., 1994), and antidepressant treatment has been shown to increase PREP serum level (Maes et al., 1995). Interestingly, here ECS treatment reduced PPCE levels in fluoxetine non-responding mice.

Finally, TALIN2, a protein involved in the cytoskeleton and the adhesion as well as in signaling pathway (by production of PIP2) increased in the serum of MDD patients (Al-Hakeim et al., 2020). Interestingly, we prior found TALIN2 to be downregulated in animals that respond to chronic fluoxetine vs. non-responding mice (Mendez-David et al., 2017). We confirmed here this association as ECS reduced TLN2 expression compared in Flx-NR mice.

A preclinical study looking at a proteomic peripheral profile of ECS effects in naïve male Sprague-Dawley rats found a biosignature that differs from what we obtained here (Glaviano et al., 2014). These discrepancies could be due to a difference in the experimental models used (naïve vs. animal model of non-response to SSRI), but also difference in the peripheral tissue studied (plasma vs. PBMC). Whether these changes observed in PBMC can correlate to similar changes in the brain remains to be tested. However, some studies shown similar expression changes between in PBMCs and the hippocampus after stress in mice (van Heerden et al., 2009), and convergent signaling pathways between amygdala and blood (Daskalakis et al., 2014). More recently, Hervé et al. (2017) have shown peripheral gene expression changes allowing for the identification of biomarkers predictive of treatment response in mice and depressed subjects. While correlation in proteomic expression between selective brain regions and PBMC has yet not been explored using high-throughput techniques, however the use of PBMCs as a surrogate for proteomic studies in psychiatric disorders has been proposed (Rahmoune and Guest 2017).

At the single molecule level, prior association of these different markers have been mentioned in the literature. However, MDD and treatment response cannot be resumed by individual factors. Thus, only association study of protein network would benefit to patients with MDD. Here, we described a main protein network based on 33 proteins involved in three regulatory pathways known to participate in the etiology of mood disorders: glucocorticoid receptor signaling (Cattaneo and Riva 2016; McEwen and Akil 2020), PI3K/Akt signaling (Fujiki et al., 2010; Matsuda et al., 2019) and NRF2-mediated oxidative stress response (Bakunina et al., 2015; Mendez-David et al., 2015). A recent review underlined biological systems related to neurotrophic and inflammatory/immune systems in the effects of ECT (Maffioletti et al., 2021), the involvement of this last system being also observed and assess in the cerebrospinal fluid in patients (Kranaster et al., 2019).

## 5 Limitations and conclusion

One of the strengths of this study is that ECS were applied in mice that did not respond to chronic fluoxetine administration. Whether this biosignature, specific to non-response to fluoxetine vs other antidepressant drug treatment, remains to be further tested. Indeed, MDD is more prevalent in women (Albert 2015). Our study was performed in male mice because we previously showed that chronic corticosterone administration in female C57BL/6 mice did not affect the emotionality score (Mekiri et al., 2017). It could be interesting to confirm whether such a biosignature could be reproduced in another animal model of anxiety/depression, e.g., chronic social defeat stress model or chronic mild stress.

Here, we tested the effects of ECS in Flx-NR animals while Flx treamtent was maintained. Thus, an interaction of ECS with Flx cannot be ruled out and the effects of ECS alone -without Flx treatment-has not be tested. However, as some clinical studies suggest, concomitant pharmacotherapy may increased ECT efficacy and decrease the risk of relapse (Sackeim et al., 2009; Haskett and Loo 2010). Similarly, we did not test whether the changes in proteins expression are state-dependent and would be similar in naïve mice, as electroconvulsive treatment are of interest in a pathological situation.

While the present study was performed in a low number of animals, we believe that its longitudinal trajectory with proteomic profiling within the same animal before and after ECS strengthen its scientific validity. This study paves the way for a greater definition of peripheral biomarkers related to antidepressant treatment response, with the goal of a better prediction and translational validation of these methods. Yet, whether or not these proteomic changes observed in PBMCs from mice mirror biological changes in brain tissues remains to be tested.

## Data Availability

The mass spectrometry proteomics data have been deposited to the ProteomeXchange Consortium via the PRIDE (Perez-Riverol et al., 2022) partner repository with the dataset identifier PXD037392.
