# Supplementary material for: Peripheral proteomic changes after electroconvulsive seizures in a rodent model of non-response to chronic fluoxetine
Source: Front Pharmacol. 2022 Oct 31;13:993449. doi: 10.3389/fphar.2022.993449 (PMC9659725; doi:10.3389/fphar.2022.993449)

Supplementary Figure 2

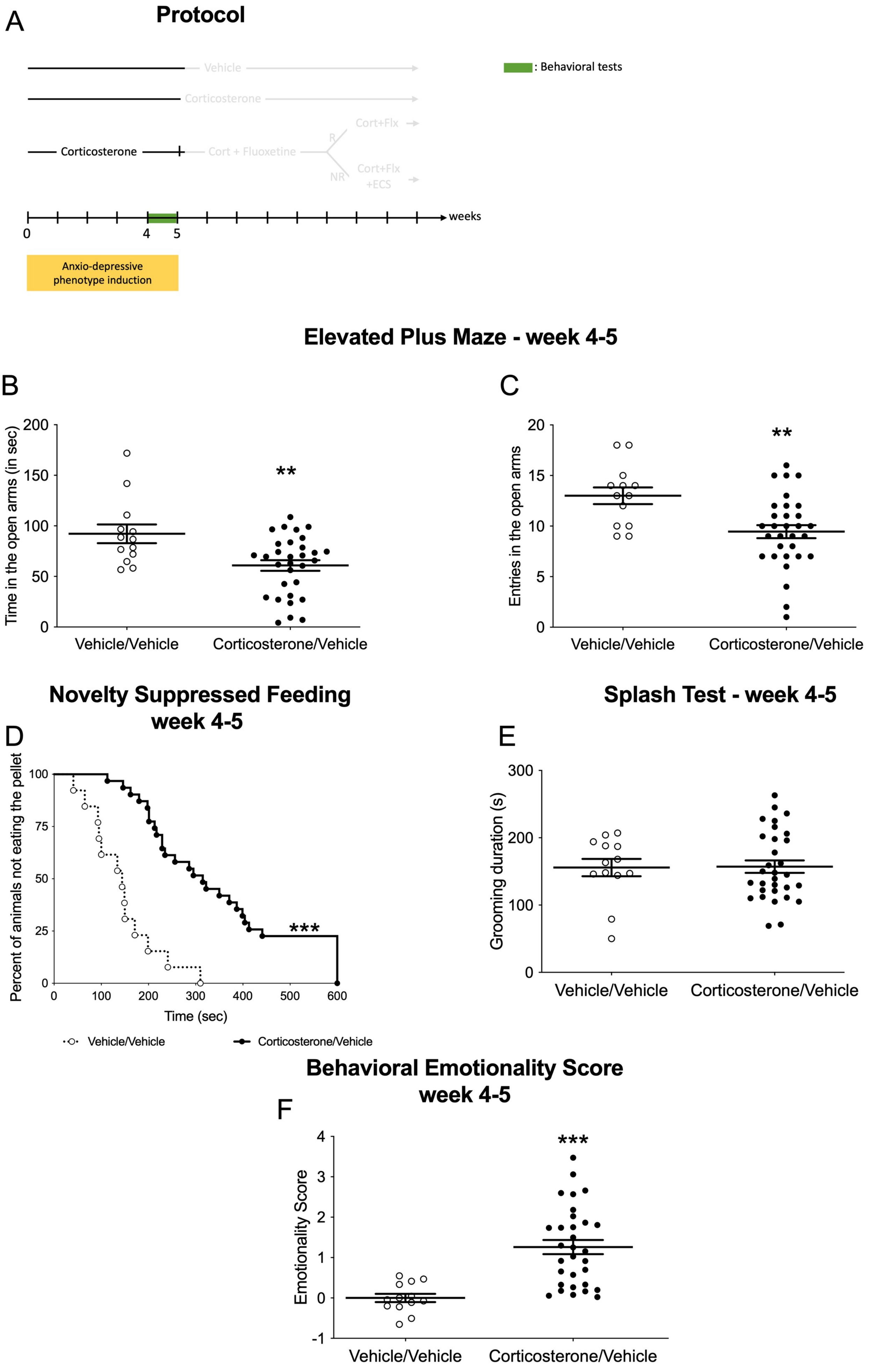

Supplement: Supplementary file 2 [file DataSheet4.PDF]
