# Supplementary material for: Peripheral proteomic changes after electroconvulsive seizures in a rodent model of non-response to chronic fluoxetine
Source: Front Pharmacol. 2022 Oct 31;13:993449. doi: 10.3389/fphar.2022.993449 (PMC9659725; doi:10.3389/fphar.2022.993449)
Supplement: Supplementary file 3 [file DataSheet3.PDF]

Supplementary Table 1: Overall statistical results

	Test	Parameter	Treatment groups number	Total number of samples	Parametric (P) or non-parametric (NP) analysis	Unpaired T-test P value	Mann Whitney test p-value	One-Way ANOVA F-value (or Kruskal-Wallis statistic)	One-Way ANOVA (or Kruskal-Wallis) P-value	Corticosterone vs Vehicle	Corticosterone+Fluoxetine (all) vs Vehicle	Corticosterone+Fluoxetine-R vs Vehicle	Corticosterone+Fluoxetine-NR vs Vehicle	Corticosterone+Fluoxetine-NR+ECS vs Vehicle	Corticosterone+Fluoxetine (all) vs Corticosterone	Corticosterone+Fluoxetine-R vs Corticosterone	Corticosterone+Fluoxetine-NR vs Corticosterone	Corticosterone+Fluoxetine-NR vs Corticosterone+Fluoxetine-R	Corticosterone+Fluoxetine-NR+ECS vs Corticosterone	Corticosterone+Fluoxetine+ECS vs Corticosterone+Fluoxetine	Log-rank (Mantel-Cox) test Chi square	Chi square P-value	Corticosterone vs Vehicle	Corticosterone+Fluoxetine (all) vs Vehicle	Corticosterone+Fluoxetine (all) vs Corticosterone	Corticosterone+Fluoxetine-R vs Vehicle	Corticosterone+Fluoxetine-NR vs Vehicle	Corticosterone+Fluoxetine-R vs Corticosterone	Corticosterone+Fluoxetine-NR vs Corticosterone	Corticosterone+Fluoxetine-NR vs Corticosterone+Fluoxetine-R	Corticosterone+Fluoxetine+ECT vs Vehicle	Corticosterone+Fluoxetine+ECT vs Corticosterone	Corticosterone+Fluoxetine+ECT vs Corticosterone+Fluoxetine	Pearson correlation in Vehicle	Pearson correlation in Corticosterone	Pearson Correlation in Corticosterone+Flx	Pearson Correlation in Corticosterone+Flx+ECT
**Supplementary Figure 1**																																					
S1A	Weight	Weight Gain at W5	2	44	P	0.004																															
S1B	Weight	Weight Gain at W10	3	44	P			8.733	0.001	0.174	0.008				0.001																						
S1C	Weight	Weight Gain at W12	4	44	P			4.182	0.012	0.047		0.010		0.645		0.932			0.034	0.010																	
**Supplementary Figure 2**																																					
S2B	Elevated Plus Maze	Time in the open arms (in sec)	2	44	P	0.003																															
S2C	Elevated Plus Maze	Entries in the open arms	2	44	P	0.003																															
S2D	Novelty Suppressed Feeding	Latency to eat the pellet	2	44	NP																26.980	<0.0001		<0.0001													
S2D (data not shown)	Novelty Suppressed Feeding	Correlation of latency and Food consumption in home cage	2	44	P	0.061																												0.4011	0.0547		
S2F	Splash Test	Grooming duration (in sec)	2	44	NP		0.689																														
S2G	Emotionality	Emotionality Score	2	44	P	<0.0001																															
**Figure 1**																																					
1B	Elevated Plus Maze	Time in the open arms (in sec)	3	44	NP			13.420	0.001	0.003	>0.9999				0.002																						
1C	Elevated Plus Maze	Entries in the open arms	3	44	P			5.135	0.010	0.021	0.482				0.003																						
1D	Novelty Suppressed Feeding	Latency to eat the pellet	3	44	P																13.360	0.001	<0.0001														
1D (data not shown)	Novelty Suppressed Feeding	Correlation of latency and Food consumption in home cage	3	44	P																													0.0773	0.9011	0.0634	
1E	Splash Test	Grooming duration (in sec)	3	44	P			3.888	0.028	0.954	0.020	0.051			0.051																						
1F	Emotionality	Emotionality Score	5	44	P			25.060	<0.0001	<0.0001	0.3067	0.0131	0.0072		<0.0001	<0.0001	0.0018	<0.0001																			
1G	Emotionality	Change in Emotionality Score	5	44	P			14.620	<0.0001	0.0984	0.0002	<0.0001	0.4908		<0.0001	<0.0002	0.4276	<0.0001																			
**Figure 2**																																					
2B	Elevated Plus Maze	Time in the open arms (in sec)	4	44	P			5.916	0.002	0.006		0.640		0.239		0.001			0.001	0.387																	
2C	Elevated Plus Maze	Entries in the open arms	4	44	P			11.600	<0.0001	0.021		0.004		0.049		<0.0001			<0.0001	0.742																	
2D	Novelty Suppressed Feeding	Latency to eat the pellet	4	44	NP																22.010	<0.0001	0.015			0.223		<0.0001			0.075	0.266	0.0003				
2D (data not shown)	Novelty Suppressed Feeding	Correlation of latency and Food consumption in home cage	4	44	P																													0.0853	0.8945	0.7112	0.681
2E	Splash Test	Grooming duration (in sec)	4	44	P			2.600	0.0651	0.068		0.325		0.882		0.008			0.138	0.141																	
2F	Emotionality	Emotionality Score	4	44	NP			19.240	0.0002	0.004		0.098		0.911		<0.0001			0.015	0.141																	
