# Supplementary material for: Peripheral proteomic changes after electroconvulsive seizures in a rodent model of non-response to chronic fluoxetine
Source: Front Pharmacol. 2022 Oct 31;13:993449. doi: 10.3389/fphar.2022.993449 (PMC9659725; doi:10.3389/fphar.2022.993449)
Supplement: Supplementary file 4 [file DataSheet5.pdf]

SUPPLEMENTARY MATERIAL

Authors: Rodolphe H. Lebeau[1], Indira Mendez-David[1], Laura Kucynski-Noyau[1], Céline Henry[2], David Attali[3], Marion Plaze[3], Romain Colle[4,5], Emmanuelle Corruble[4,5], Alain M. Gardier[1], Raphaël Gaillard[3], Jean-Philippe Guilloux[*,1,§], Denis J. David[*,1].

Short Title: Proteomic changes after ECS

[1] Batiment Henri Moissan, Université Paris-Saclay, CESP-Inserm, MOODS Team, Orsay, 91400 France.

[2] PAPPSO, Micalis Institute, INRAE, AgroParisTech, Université Paris-Saclay, 78350 Jouy-en-Josas, France.

[3] Centre Hospitalier Sainte Anne, Service Hospitalo-Universitaire, Paris, France

[4] Université Paris-Saclay, Faculté de Médecine, CESP-Inserm, MOODS Team, Le Kremlin Bicêtre, F-94275, France.

[5] Service Hospitalo-Universitaire de Psychiatrie de Bicêtre, Hôpitaux Universitaires Paris-Saclay, Assistance Publique-Hôpitaux de Paris, Hôpital de Bicêtre, Le Kremlin Bicêtre, F-94275, France.

* co-last authorship

[§] To whom correspondence should be sent:

Dr Jean-Philippe Guiloux
Inserm, CESP, Equipe « Moods »
Université Paris Saclay - Batiment Henri Moissan
19 avenue des Sciences
91400 Orsay France
Tel : +33.(0)1.80.00.63.35
jean-philippe.guilloux@universite-paris-saclay.fr

LEGENDS

Supplemental Table 1: Overall statistical results for behavioral studies

Supplemental Table 2: Proteins significantly differentially expressed after one-way ANOVA. Protein ID: A single reference to the protein in this grouping experiment (unique within a sample in individual mode); Cort/Veh: Corticosterone/Vehicle; Cort/Flx-NR: Corticosterone/Fluoxetine non-responder; Cort/Flx-ECS: Corticosterone/Fluoxetine after receiving ECS.

Supplemental Figure 1: Change in mouse body weight across the protocol.
In place of normal drinking water, grouped-housed male C57BL/6JRj mice were presented during 10 weeks with vehicle (0.45% hydroxypropyl-β-cyclodextrin) or corticosterone (35 μg/ml) in the presence or absence of an antidepressant (fluoxetine, 18 mg/kg/day) during the last five weeks of the corticosterone regimen. Mouse body weigh was followed after corticosterone regimen (**A**) (W5), after corticosterone regimen in presence or absence of fluoxetine (**B**) (W10) and following electroconvulsive seizures in fluoxetine non-responder animals (**C**).
Values plotted are means ± SEM [n = 7-31 animals depending on the group/time of measurement]. Group differences were monitored using Student t-Test (**A**) or one-way ANOVA with post-hoc tests (**B** and **C**) *p<0.05, **p<0.01 *versus* Vehicle/vehicle group, #p<0.05, ###p<0.001 *versus* Cort/vehicle group, §§p<0.01 *versus* Flx-R mice.

Supplemental Figure 2: A 4-week corticosterone treatment (35 μg/ml) induced an anxiety/depression-like phenotype in C57BL/6JRj mice.
(A) Timeline of experiments: In place of normal drinking water, grouped-housed male C57BL/6JRj mice were presented during 10 weeks with vehicle (0.45% hydroxypropyl-β-cyclodextrin) or corticosterone (35 μg/ml) Then, we investigated whether the behavioral changes induced after chronic corticosterone (week 4 to 5, figure S2B-F)**. (B,C)** Effects of corticosterone (35 μg/ml, Cort) regimen on anxiety behavior in the Elevated Plus Maze (EPM). Anxious behaviour was monitored using mean total time in seconds **(B)** or entries **(C)** in the open arms of the EPM paradigm. **(D)** Effects of 4 weeks of corticosterone regimen on anxiety- and depression related behaviors in the Novelty Suppressed Feeding paradigm. Results are expressed as cumulative survival with percentage of animals that have not eaten over 10-min. **(E)** Effects of 4 weeks of corticosterone regimen on grooming behavior in the Splash Test (ST). Results are expressed as mean of grooming duration (in seconds). **(F)** Effects of 4 weeks of

corticosterone regimen on anxiety/depression-like behaviors resumed in the emotionality score. Values plotted are means ± SEM [n = 7-31 animals depending on the group/time of measurement]. Unpaired *t*-test (*p<0.05, **p<0.01, ***p<0.001 *versus* Vehicle/vehicle group) or Kaplan–Meier survival analysis followed by Mantel–Cox log-rank test were applied (**p<0.01 *versus* Vehicle/vehicle group,).
